# Integrating Artificial Intelligence in Next-Generation Sequencing: Advances, Challenges, and Future Directions

**DOI:** 10.3390/cimb47060470

**Published:** 2025-06-19

**Authors:** Konstantina Athanasopoulou, Vasiliki-Ioanna Michalopoulou, Andreas Scorilas, Panagiotis G. Adamopoulos

**Affiliations:** Department of Biochemistry and Molecular Biology, Faculty of Biology, National and Kapodistrian University of Athens, 15701 Athens, Greece; konnath@biol.uoa.gr (K.A.); vasilinamichalopoulou@gmail.com (V.-I.M.); ascorilas@biol.uoa.gr (A.S.)

**Keywords:** NGS, AI, machine learning, deep learning, genomics, transcriptomics, precision medicine, cancer research, drug discovery, data integration

## Abstract

The integration of artificial intelligence (AI) into next-generation sequencing (NGS) has revolutionized genomics, offering unprecedented advancements in data analysis, accuracy, and scalability. This review explores the synergistic relationship between AI and NGS, highlighting its transformative impact across genomic research and clinical applications. AI-driven tools, including machine learning and deep learning, enhance every aspect of NGS workflows—from experimental design and wet-lab automation to bioinformatics analysis of the generated raw data. Key applications of AI integration in NGS include variant calling, epigenomic profiling, transcriptomics, and single-cell sequencing, where AI models such as CNNs, RNNs, and hybrid architectures outperform traditional methods. In cancer research, AI enables precise tumor subtyping, biomarker discovery, and personalized therapy prediction, while in drug discovery, it accelerates target identification and repurposing. Despite these advancements, challenges persist, including data heterogeneity, model interpretability, and ethical concerns. This review also discusses the emerging role of AI in third-generation sequencing (TGS), addressing long-read-specific challenges, like fast and accurate basecalling, as well as epigenetic modification detection. Future directions should focus on implementing federated learning to address data privacy, advancing interpretable AI to improve clinical trust and developing unified frameworks for seamless integration of multi-modal omics data. By fostering interdisciplinary collaboration, AI promises to unlock new frontiers in precision medicine, making genomic insights more actionable and scalable.

## 1. Introduction

The 20th century marked a pivotal revolution in our understanding of the genetic foundations of life. The discovery of the double-helix structure of DNA and the elucidation of the central dogma of molecular biology laid the groundwork for a new era in human biology and genomics [1,2]. These discoveries redefined biology as a science fundamentally driven by the genetic alphabet of adenine, thymine, cytosine, and guanine. However, deciphering this “book of life” required not only biochemical ingenuity but also significant technological innovation. This transition was accelerated by the completion of the Human Genome Project (HGP), which catalyzed rapid advances in sequencing technology and computational methods, ultimately reshaping the landscape of genomic research [3,4]. Despite the early contributions of Sanger sequencing, the HGP completion did not mark an endpoint but, rather, the beginning of a new era, unveiling the complexity of the human genome and highlighting the limitations of manual data analysis [5].

Following the completion of the HGP, sequencing technologies demonstrated significant improvements at an exponential rate. Next-generation sequencing (NGS) and third-generation sequencing (TGS) platforms, such as those developed by Illumina^®^ and Oxford Nanopore Technologies (ONT), respectively, revolutionized genomic data acquisition by offering high-throughput and cost-effective sequencing solutions. These innovations enabled the sequencing of entire genomes within hours, significantly reducing costs and accelerating both research and clinical applications [6]. Concurrently, genome editing tools evolved remarkably, with the CRISPR/Cas9 system transforming genome editing from a theoretical concept into a precise and programmable tool. This breakthrough enabled targeted manipulations of DNA sequences with unprecedented versatility and accuracy, revolutionizing genetic research and therapeutic development [7].

Furthermore, the genomic data volume, and thus the amount of information per experiment, has increased exponentially, surpassing in many cases the capabilities of traditional computational approaches. Despite these advances, NGS data analysis continues to face major challenges. These include the sheer volume of sequencing data, the complexity and variability of biological signals, and the prevalence of technical artifacts such as amplification bias, batch effects, and sequencing errors. Traditional computational tools often struggle with these issues, which motivates the integration of artificial intelligence (AI) approaches that can model nonlinear patterns, automate feature extraction, and improve interpretability across large-scale datasets. This bottleneck catalyzed the adoption and integration of AI into genomics. Machine learning (ML) and deep learning (DL) algorithms, powered by advancements in neural networks and cloud computing, emerged as indispensable tools. Advanced DL models are now being employed to predict the structural and functional characteristics of genes from DNA or protein sequences, revealing intricate patterns beyond human discernment and effectively bridging the gap between data generation and biological interpretation [8].

Today, AI and genomics operate within a dynamic feedback loop. AI enhances genomic research by streamlining experimental design, simulating outcomes through predictive models, automating laboratory procedures to reduce manual work, and facilitating complex data analysis. Conversely, the vast repositories of genomic data enhance AI systems, refining their capacity to emulate complex biological reasoning. However, this synergy also raises significant ethical concerns, including cognitive offloading, algorithmic biases, privacy issues, as well as the profound moral implications of editing the fundamental code of life. These challenges necessitate urgent and ongoing discourse [9,10].

In this review, we explore the synergistic interplay between AI and genomics, with a focused exploration on AI’s contributions to NGS applications and data analysis. We highlight AI’s transformative impact across multiple NGS workflows—from experimental design to automated library preparation and implementation of AI-driven pipelines. Special attention is given to how ML enhances the accuracy of NGS data interpretation, enables predictive modeling of editing outcomes, and accelerates the discovery of novel findings. The discussion extends to current challenges in AI-assisted genomic analysis, including scalability, bias mitigation, and ethical considerations in precision genome editing. Through this discussion, we aim to highlight the capabilities of AI-based tools that can be utilized to overcome impediments in NGS-based research and clinical applications.

## 2. AI in the Laboratory: Enhancing Research from Plan to Data Analysis

In recent years, the convergence of AI and genomics has garnered significant academic and research attention, as well as substantial investments from universities, research institutions, hospitals, and pharmaceutical companies. This integration has fully transformed the landscape of genomic research, incorporating AI-driven methodologies—including ML and DL—that enhance our capacity to analyze complex biological data, yielding more accurate results [11,12]. This evolution represents a paradigm shift in how genomic information is interpreted and applied, reflecting AI’s transformative impact across the field of sequencing.

### 2.1. The Pre-Wet-Lab Phase

**Figure 1:**
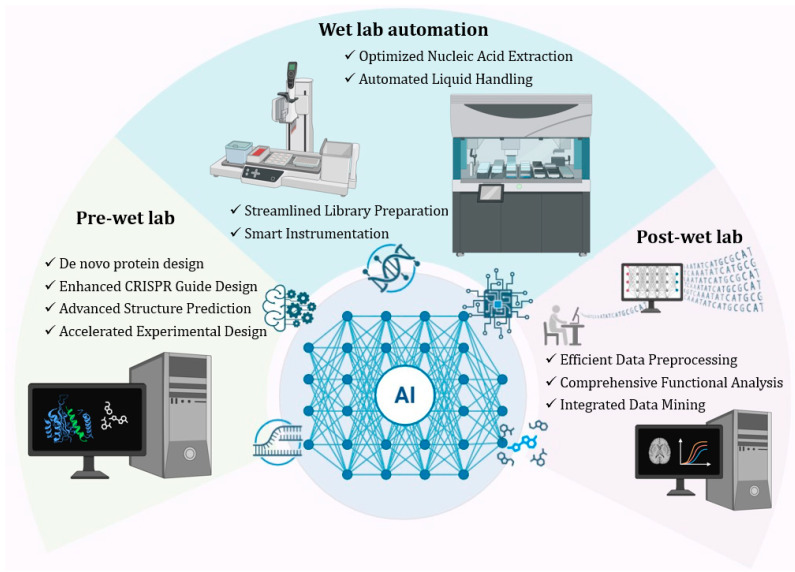
Artificial intelligence enhances NGS-based workflows across pre-wet lab, wet-lab automation, and post-wet-lab phases.

The pre-wet-lab phase in genomics has traditionally been characterized by manual experimental design, which was heavily reliant on prior knowledge, empirical guidelines, and trial-and-error methods. With the advent of AI, pre-wet-lab procedures have undergone a radical transformation [13]. Today, AI-driven computational tools play a pivotal role in the strategic planning of experiments, assisting researchers in predicting outcomes, optimizing protocols, and anticipating potential challenges prior to initiating wet-lab work (Figure 1).

Briefly, several AI tools can be used to enhance the pre-wet-lab phase by improving experimental design and simulating outcomes. Benchling is a cloud-based platform that integrates AI to help researchers efficiently design experiments, optimize protocols, and manage lab data [14]. Similarly, DeepGene, an AI-powered tool, uses advanced deep neural networks to predict gene expression and assess experimental conditions, offering insight into the expected outcomes before laboratory work begins [15]. Of note, platforms such as Labster provide interactive virtual labs that simulate experimental setups, enabling researchers to visualize outcomes and troubleshoot potential failures in a risk-free environment. In addition, tools, including Indigo AI (https://www.fintechscotland.com/fintech/indigo-ai/, accessed on 16 June 2025) and LabGPT (https://chatgpt.com/g/g-3eIYfoFVJ-labgpt, accessed on 16 June 2025), offer generative AI capabilities for automated protocol generation and experimental planning, while Synthace enables AI-powered protocol optimization and lab automation. Colabra supports collaborative planning and experiment tracking using AI-enhanced project workflows, and DeepChem offers ML frameworks for molecular property prediction, aiding compound screening and hypothesis formulation. These AI-driven innovations not only improve the efficiency of the experimental design process but also help to identify and mitigate potential issues before they arise in the wet lab, thus enhancing the overall success of genomic research.

### 2.2. The Wet-Lab Phase

AI’s impact extends into the wet-lab phase, transforming laboratory workflows through automation, optimization, and real-time analysis. AI-driven automation technologies have streamlined traditional labor-intensive procedures, significantly improving reproducibility, scalability, and data quality (Figure 1). For instance, the Tecan Fluent systems are highly modular, deck-based liquid handling workstations that can be tailored to automate plate- or tube-based assays [16]. These platforms are particularly effective in automating tasks such as PCR and qPCR setup, NGS library preparation, nucleic acid extractions, and CRISPR workflows, utilizing AI algorithms to detect worktable and pipetting errors [17]. More precisely, in CRISPR workflows, AI-powered platforms have emerged to streamline both experimental design and validation. For instance, Synthego’s CRISPR Design Studio offers automated gRNA design, editing outcome prediction, and end-to-end workflow planning, while tools like DeepCRISPR use DL to maximize editing efficiency and minimize off-target effects [18]. Additionally, R-CRISPR is used for gRNA design and combines convolutional neural networks (CNNs) and recurrent neural networks (RNNs) to predict off-target effects with mismatch or indels [19]. In proteomics and immunoassay workflows, these platforms facilitate the automation of ELISA, bead-based immunoassays, as well as protein digestion for mass spectrometry. Additionally, in cell-based assays, the systems can handle automated cell seeding, cell culture media change, staining and fixing steps, and cytotoxicity assays [20].

Beyond automation, AI enhances laboratory workflows through real-time monitoring and feedback control. For instance, a recent study integrated the AI-powered YOLOv8 model with the Opentrons OT-2 liquid handling robot to provide real-time quality control. This system enables precise detection of pipette tips and liquid volumes, offering immediate feedback to correct errors, including missing tips or incorrect liquid volumes, thereby ensuring experimental accuracy [21]. Such AI-driven solutions can significantly facilitate laboratory automation, making advanced capabilities accessible even in resource-limited settings.

### 2.3. The Post-Wet-Lab Phase

Following experimental implementation, the post-wet-lab phase has traditionally involved intensive and complex data analysis, a process frequently hindered by the complexity of genomic datasets (Figure 1). AI has dramatically accelerated this phase by providing tools that streamline bioinformatics and enhance data interpretation. Platforms such as Illumina BaseSpace Sequence Hub and DNAnexus enable bioinformatics analyses without requiring advanced programming skills or command-line tools. These cloud-based environments have recently incorporated bioinformatics tools that leverage AI/ML to perform analysis of complex genomic and biomedical data. Their user-friendly graphical interfaces often support custom pipeline construction through intuitive drag-and-drop features. DL models trained on large-scale biological datasets can identify subtle patterns, predict biological functions, and suggest mechanistic hypotheses that might elude traditional statistical approaches. Tools like DeepVariant apply deep neural networks to improve the accuracy of variant calling from sequencing data, surpassing traditional heuristic-based approaches [22]. As for CRISPR post-experiment analysis, tools such as CRISPResso2, GUIDE-seq, and Cas-OFFinder are widely used to detect and quantify genome-wide off-target edits [23,24,25]. More advanced methods, including CRISPRitz and AI-enhanced scoring models in DeepCRISPR, provide flexible and rapid off-target prediction, improving both accuracy and safety in gene-editing applications [26,27].

The availability of extensive online biological databases has become essential to post-wet-lab research. Public repositories, such as NCBI’s Gene Expression Omnibus (GEO), ENCODE, TCGA, and EMBL-EBI, provide access to large-scale, curated datasets across genomics, transcriptomics, and epigenomics [28,29,30,31]. AI and computational platforms now assist in dataset selection and functional comparison. OMICtools provides a searchable catalog of bioinformatics tools and knockout-specific datasets [32]. MAGeCK enables normalization, ranking, and comparison of gene dependencies from pooled CRISPR screens across multiple cell lines [33]. Graph-based systems like KnetMiner support gene-centric queries, retrieving knockout experiments, and comparing affected pathways and phenotypes across species [34]. After dataset retrieval, analysis systems such as ShinyGO, ReactomeGSA, and Metascape facilitate cross-study enrichment and network comparison, revealing shared and divergent biological impacts of gene knockouts in various models [35,36,37].

Moreover, novel AI-driven discovery frameworks are now applied to gene regulatory networks (GRNs). These tools use curiosity-driven exploration algorithms to probe the range of stable “goal states” that GRNs can robustly reach—effectively mapping a “behavioral catalog” for each network. For instance, in one study spanning 432 GRNs from plants, bacteria, rodents, and humans, natural networks exhibited orders of magnitude higher behavioral versatility than matched random networks [38]. Additionally, MICRAT is a specialized algorithm for inferring GRNs from time-series gene expression data, enhancing directionality and causal insight by combining MIC with conditional entropy and temporal information [39]. These tools enable quantitative comparisons of network robustness and versatility across organisms and can identify clinically relevant states and facilitate therapeutic strategy design.

AI-driven search and meta-analysis tools enhance the utility of these databases by enabling cross-study comparisons, predicting functional associations, and detecting subtle patterns that may not be apparent through conventional methods [40]. Recently, the generative AI models of ChatGPT (GPT-4o), DeepSeek (version V3), and BioGPT (https://github.com/microsoft/) have emerged as powerful tools for scientific interpretation and experimental design refinement [41,42,43]. These language models assist researchers in interpreting complex datasets, summarizing extensive experimental results, and proposing biological insights based on multi-omics data. However, while AI performs highly sophisticated analyses, it is crucial to validate findings through orthogonal methods and maintain a critical perspective. Automated interpretation, although powerful, is not immune to biases or data artifacts, emphasizing the need for human oversight.

## 3. AI in NGS Data Analysis

The analysis of NGS data has been radically transformed by the application of AI models, which provide powerful tools for pattern recognition, classification, and exploratory analysis. These approaches are particularly valuable for their interpretability and computational efficiency, especially in cases of genomic and transcriptomic datasets. To efficiently manage and retrieve specific output files from large-scale NGS datasets, several workflow management systems and automation tools have been developed (Table 1). Nextflow is one such widely adopted workflow management system that enables scalable, reproducible data analysis across diverse computing environments. It streamlines complex pipelines by automating file handling, software dependencies, and output organization using features like the publishDir directive [44].

### 3.1. Machine Learning Approaches

Supervised ML algorithms have become essential for NGS applications requiring precise classification or regression. Random forests (RFs) and support vector machines (SVMs) represent the most widely adopted methods due to their robustness against overfitting and their ability to handle high-throughput sequencing data. In variant calling, RF-based pipelines, like GATK (© Broad Institute, USA) (Genome Analysis Toolkit), demonstrate superior performance in distinguishing true genetic variants from sequencing artifacts by integrating multiple quality metrics [58,59]. Gradient boosted machines (GBMs) have also proven effective for predicting variant pathogenicity when trained on curated clinical datasets [60]. For transcriptomic data, supervised learning enables disease subtyping and biomarker discovery [61]. The PAM50 classifier, which uses an SVM-based approach to identify breast cancer subtypes from RNA-Seq data, has been widely known as a clinical standard [62]. Similarly, ML models have been successfully applied to develop prognostic signatures for various cancers by selecting target genes from expression profiles [63].

Unsupervised techniques are indispensable for extracting patterns from unlabeled NGS data. Principal component analysis (PCA), while a statistical rather than a classical ML technique, is widely used for dimensionality reduction in genomic datasets to implement clustering and visualization. PCA-based tools enable fast and memory-efficient analysis of single-nucleotide polymorphism (SNP) variations in population-scale studies [64]. Notably, more advanced methods, such as t-distributed stochastic neighbor embedding (t-SNE) [65] and uniform manifold approximation and projection (UMAP) [66], facilitate the visualization of high-dimensional datasets. These techniques have proven transformative in single-cell RNA sequencing (scRNA-seq) studies, enabling the discovery of novel cell states and populations.

While ML methods offer advantages in interpretability and present relatively low computational requirements, they can be limited in handling high-dimensional NGS data. Feature selection becomes critical to mitigate the “curse of dimensionality,” and for more complex applications, integration with DL techniques may offer improved performance. Finally, careful attention must be paid to batch effects and technical artifacts, which can confound ML-driven analyses if not properly examined.

### 3.2. Deep Learning Approaches

DL architectures such as CNNs, RNNs, and transformers are particularly well-suited for NGS data due to their ability to model distinct data characteristics. CNNs excel at identifying localized patterns indicative of mutations or sequence motifs, making them ideal for tasks such as variant calling and transcription factor binding site prediction [67]. RNNs, including long short-term memory networks (LSTMs), capture sequential dependencies and are powerful for modeling long genomic sequences. Transformer-based models leverage self-attention mechanisms to capture both local and long-range dependencies efficiently, which is critical for interpreting regulatory elements and 3D genomic interactions.

DL has emerged as a powerful approach in NGS data analysis, addressing key limitations of traditional machine learning by automatically extracting hierarchical representations from raw, high-dimensional data [68]. Neural network architectures eliminate the need for manual feature engineering and exhibit exceptional capabilities in modeling complex, nonlinear biological interactions. CNNs have become fundamental in sequence-based analyses due to their ability to capture local sequence motifs [69,70]. Early applications demonstrated CNNs’ effectiveness in predicting transcription factor binding sites directly from DNA sequences [71,72]. In clinical genomics, CNN-based variant calling models have substantially improved performance by interpreting sequencing reads as image-like data, exhibiting notably higher accuracy than traditional heuristic methods [73]. Furthermore, CNN architectures have been utilized to identify the functional effects of non-coding variants by predicting chromatin accessibility across hundreds of cell types and annotating disease-associated SNPs [74].

RNNs, particularly LSTMs, have been employed to genomic sequence data analysis due to their ability to capture sequential dependencies and long-range interactions [75]. RNNs are designed to capture time-series data or sequence information, making them powerful tools for multiple genomic and transcriptomic tasks that include chromatin accessibility analysis [76], identification of RNA splicing events [75], variant interpretation [77], and metagenomic analysis [78]. LSTMs, a type of RNN, have been specifically designed to address the issue of vanishing gradients by maintaining long-term dependencies, which is critical when analyzing long genomic sequences. LSTM models have been successfully employed to predict splicing alterations in RNA sequences, enabling the identification of pathogenic variants in rare diseases with remarkable accuracy [79]. Bidirectional LSTMs (BLSTMs) model long-range dependencies in nucleotide sequences by processing them in both forward and reverse directions using gating mechanisms that retain contextual memory [77]. They are widely used in tasks such as transcription factor binding prediction and variant calling, often in combination with CNNs, as exemplified by the DAVI model [70,80].

Beyond basic sequence modeling, RNNs with attention mechanisms have further improved the interpretability and performance of multiple models. Attention mechanisms allow the model to focus on the most relevant parts of the sequence, making it more efficient at predicting complex biological phenomena. One noteworthy application is the prediction of gene expression based on histone modification patterns, where RNNs with attention mechanisms outperformed traditional classifiers in terms of AUC (area under the curve) [81]. The attention-based approach helps identify key regulatory regions that are crucial for understanding gene regulation, providing a more refined model for genomic analysis.

### 3.3. Hybrid and Ensemble Approaches

Hybrid and ensemble methods address NGS challenges by strategically combining algorithms or data modalities [82]. In the context of genomics, hybrid models often integrate ML and DL methods to manage the complexity of high-dimensional data and overcome drawbacks inherent in single-algorithm models. Hybrid CNN-RNN approaches are employed for sequence-based tasks where both local motifs and long-range dependencies are important, such as variant calling and splicing prediction. Indicatively, Clair3 [83] and PEPPER-Margin-DeepVariant [84] represent hybrid architectures by combining CNN-based feature extraction with sequence modeling for long-read variant detection. Ensemble methods, on the other hand, aggregate predictions from multiple base learners—such as decision trees, support vector machines, or neural networks—using techniques like bagging, boosting, or stacking. Ensemble techniques like XGBoost-RF hybrids (e.g., in ClinPred [85]) integrate tree-based models to improve pathogenic variant classification, outperforming single-model approaches in clinical datasets. These ensemble models significantly outperform individual algorithms in tasks like missense variant interpretation and cancer gene prioritization. Although these approaches increase computational complexity, they provide a pathway towards more accurate and generalizable AI models, which are essential for the efficient clinical translation of genomic analyses.

### 3.4. Deploying AI Within Clinical NGS Workflows

The integration of AI into clinical NGS workflows presents several challenges. Data heterogeneity, including batch effects and class imbalance in rare disease datasets, significantly reduces model accuracy and reliability [86]. Moreover, the substantial computational requirements—such as high GPU memory and large-scale storage for whole-genome data—pose logistical constraints. Reproducibility remains a persistent limitation, with many models exhibiting reduced performance upon external validation. Variability across sequencing platforms further compromises generalizability. Emerging solutions like federated learning offer improved cross-institutional performance, while cloud-based APIs help reduce local computational demands. Continuous learning systems that incorporate clinician feedback enhance adaptability, though they address only part of the deployment gap. Ultimately, aligning research with clinical application will require advances in model transparency, standardization, and infrastructure.

## 4. AI in NGS Applications

**Figure 2:**
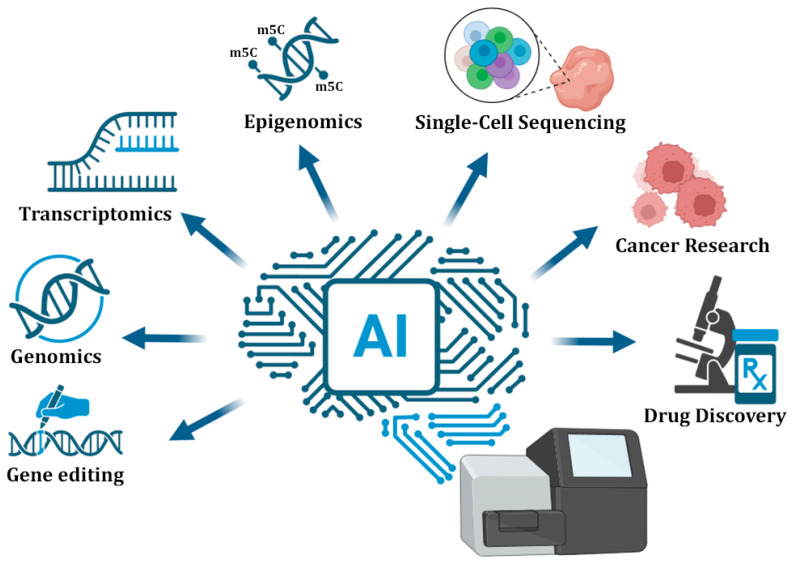
The integration of AI into NGS applications for both research and clinical purposes. AI has revolutionized the landscape of gene editing approaches, genomics, epigenomics, transcriptomics, as well as clinical applications, including cancer research and drug discoveries.

The integration of AI into NGS applications is revolutionizing the landscape of genomics, epigenomics, transcriptomics, and clinical diagnostics (Figure 2). By leveraging advanced techniques such as NNs and language models, AI enables the classification of raw sequencing data with unprecedented precision. This allows for more accurate variant detection, comprehensive functional annotation of complex genomic regions, as well as the discovery of robust biomarkers. In oncology, AI-driven NGS analysis can efficiently contribute to tumor subtyping and therapy selection, while in the context of rare diseases, ML enhances the prioritization of pathogenic variants from whole-genome sequencing data. Furthermore, the fusion of multi-omics data strengthens predictive models, advancing personalized medicine by offering deeper insights into disease mechanisms and treatment responses.

### 4.1. Genomics and Epigenomics

In the last few years, AI-powered genomic analysis has become indispensable for interpreting the functional impact of genetic variants and enabling precision medicine. Firstly, the process of identifying genetic variants from NGS datasets has been significantly enhanced by AI-based tools, since they offer improved accuracy, efficiency, and scalability as compared to traditional statistical methods. Two noteworthy DL models, DeepVariant [22] and DeepFilter [87], apply CNNs to distinguish mutations from sequencing artifacts in challenging genomic regions, including homopolymers and segmental duplications, significantly improving accuracy. DeepVariant reduces false positives by analyzing sequencing reads as pileup images, while DeepFilter enhances the precision of VarDict calls by postprocessing labeled variant data. For pathogenicity prediction, ensemble methods integrate evolutionary conservations, protein structures, and clinical annotations. Another alternative and very recent approach for variant calling is DNAscope, which combines GATK’s HaplotypeCaller with an AI-based genotyping model for efficient processing of large datasets [88]. Additionally, frameworks such as REVEL aggregate outputs from multiple individual predictors using RFs, achieving high accuracy for deleterious missense variants [89]. Recent transformer-based models like DNABERT capture long-range dependencies to interpret non-coding variants, identifying pathogenic regulatory mutations in previously undiagnosed rare disease cases [90].

In addition to genomics, AI has radically transformed epigenomic analysis by enabling accurate and scalable prediction of DNA and/or RNA methylation patterns, chromatin accessibility, and histone modification dynamics from sequencing data. In the context of chromatin state analysis, tools like ChromHMM use histone mark profiles and hidden Markov models to segment the genome into active (euchromatin) and repressive (heterochromatin) regions across various cell types [91,92]. More recently, DL frameworks such as EPInformer have leveraged DL to predict chromatin accessibility from DNA sequences, enabling insights into chromatin state dynamics [93]. Additionally, DeepChrome and DeepDiff use CNNs and LSTMs, accordingly, to integrate histone modification data and predict gene expression, offering indirect inference of chromatin openness [94,95].

DL models leverage CNNs and RNNs to capture sequence context and long-range dependencies, outperforming traditional statistical methods and thus enhancing the identification accuracy of CpG methylation at single-base resolution [96,97]. Of note, the AI-driven DNA methylation tool DeepMethyl [98] analyzes whole-genome bisulfite sequencing (WGBS) data to identify differentially methylated regions (DMRs) with increased sensitivity in cancer epigenomes. In addition, MethylNet and MethylSPWNet further extend this capability by incorporating multi-layer perceptrons and Bayesian priors for improved generalization across sample types [99,100]. It should be noted that among the most recent advancements are MethylGPT, a foundation model for the DNA methylome, and DiffuCpG, a cutting-edge generative AI model designed to address missing data in high-throughput methylation studies. MethylGPT stands out for its ability to integrate complex DNA methylation data, while DiffuCpG is particularly effective in leveraging both short- and long-range interactions, including three-dimensional genome architecture, to improve dataset accuracy, scalability, and versatility [96,97]. Both models represent novel strides in epigenetic research, with DiffuCpG demonstrating substantial improvements across multiple tissue types, cancers, and sequencing technologies (Table 2).

Chromatin accessibility prediction has progressed significantly with DL models such as Basset [101], which applies CNNs to associate DNA sequence motifs with open chromatin regions from ATAC-seq and DNase-seq data. More recently, transformer-based models, such as Enformer, have outperformed earlier architectures by capturing long-range genomic interactions, enhancing the prediction of distal enhancers and regulatory elements [102]. In single-cell epigenomics, tools like scATAC-pro employ variational autoencoders and probabilistic graphical models to resolve cell-type-specific accessibility in complex tissues [103] (Table 2). Moreover, scGraphformer represents a graph-transformer-based model that captures both local chromatin accessibility and global regulatory interactions in single-cell ATAC-seq data, enabling accurate cell-type annotation and regulatory network reconstruction [104].

Regarding histone modification analysis, a recent tool called DeepHistone integrates ChIP-seq profiles and DNA sequence features to predict the genome-wide distribution of canonical histone marks [105]. Recently, the transformer-based model dHICA introduced another robust framework for accurate histone mark imputation from chromatin accessibility data, demonstrating how AI can infer missing epigenomic layers and uncover gene regulatory relationships [106].

At the level of 3D genome organization, generative models are emerging as powerful tools. ChromoGen, a diffusion-based model, predicts single-cell chromatin conformations by learning from paired chromatin accessibility and genomic contact data, addressing variability and sparsity in single-cell 3D genome measurements [107] (Table 2). A broader review of DL approaches also highlights AI’s growing role in decoding chromatin interactions and integrating diverse epigenomic profiles for comprehensive regulatory inference [108]. Collectively, these advancements demonstrate how AI-driven tools are reshaping our understanding of the chromatin landscape at both bulk and single-cell resolution [109].

### 4.2. Transcriptomics

AI has already been widely integrated in transcriptomic analysis by enabling precise identification of gene expression patterns and alternative splicing events from RNA-Seq data, offering high-resolution snapshots of mRNA exon–intron boundaries and expression. In addition to the fact that up until today, noise, batch effects, and complex experimental designs often hinder accurate interpretation, DL models have provided innovative solutions across the transcriptomics workflow—from normalization and feature selection to differential expression analysis (DEA) and splicing prediction [110]. CNNs outperform traditional statistical methods in detecting subtle expression changes, particularly in low-abundance transcripts. A newly introduced tool, deep count autoencoder (DCA) applies deep autoencoder networks to denoise and compress gene expression data, improving downstream analyses and visualization [111]. AI models, particularly DL architectures, have shown remarkable success in cell type and subtype identification based on scRNA-seq data. Both scVI and scVAE represent two recent variational autoencoders capable of correcting batch effects, while preserving biological variation, enabling large-scale atlas integration, unsupervised clustering, trajectory inference, and DEA with minimal manual intervention [112,113,114] (Table 2). Additionally, numerous CNN-based tools and/or frameworks have been developed to predict the functional impact of non-coding variants on gene expression and chromatin features, including DeepSEA [72], Basset [101], and DeepFIGV [115].

The limited accuracy of short-read alignment is overcome by graph neural networks (GNNs), which are effective in modeling transcriptome complexity, enabling improved reconstruction and quantification. Tools such as Bambu utilize splice graph representations to reconstruct full-length transcripts with high precision, significantly outperforming traditional linear models in capturing isoform diversity [116]. For alternative splicing analysis, SpliceAI [117,118,119] and Splam employ DL models trained directly on raw genomic sequences to predict branchpoints and splice variants directly from sequence context with exceptional performance [120].

### 4.3. Single-Cell Sequencing

AI plays a pivotal role in advancing the field of single-cell sequencing analysis, addressing the unique challenges associated with this approach. ML techniques are extensively used for various aspects of single-cell sequencing data, including imputing missing gene expression values often caused by technical noise, removing batch effects that arise from processing samples at different times or in different laboratories, identifying distinct cell types and their states, estimating copy number variations within individual cells, inferring cellular differentiation trajectories, analyzing cell–cell interactions, and reconstructing regulatory networks [121]. These advances are driven by three key applications: cell type identification, trajectory inference, and heterogeneity analysis.

Cell type identification benefits from NNs that integrate gene expression with epigenetic data. scANVI, a semi-supervised extension of the variational autoencoder framework, enables precise annotation of rare and ambiguous cell types with noteworthy precision, even in minimally labeled datasets. Multi-modal models like TotalVI, which utilize CITE-seq data, can further improve classification accuracy, particularly in immune profiling tasks [122] (Table 2). Trajectory inference has advanced through neural ordinary differential equations (ODEs) and generative models [123]. Deep generative models incorporating neural ODEs reconstruct branching and cyclic trajectories, capturing progenitor states previously undetected in a subset of datasets [124,125]. Among the analysis solutions AI can offer today, scShaper offers benchmarking for many trajectory inference algorithms [126], while CopyKAT infers large-scale copy number alterations and malignant clones from scRNA-seq with high concordance to DNA sequencing [127] (Table 2).

### 4.4. Cancer Research

AI-based NGS analysis is transforming cancer research by enabling precise molecular subtyping, biomarker discovery, and personalized treatment strategies [128]. DL models now classify tumor subtypes with high accuracy using whole exome sequencing data, outperforming traditional pathology in multicenter validations [129]. Tumor heterogeneity analysis particularly benefits from AI tools that resolve clonal architecture from bulk and single-cell sequencing [130]. PyClone-VI, which employs variational Bayesian inference, detects resistant clones present at extremely low frequencies [131] (Table 2). Graph neural networks have been applied to reconstruct phylogenetic trees of tumor progression, showing high concordance with longitudinal clinical data and revealing metastatic drivers in a substantial subset of analyzed cases [132]. In the single-cell context, HoneyBADGER allows simultaneous analysis of copy number variation and gene expression to identify genetic subclones with distinct transcriptional programs, as demonstrated in progressive multiple myeloma [133]. While HoneyBADGER does not utilize DL or neural networks, it integrates probabilistic modeling and statistical inference—core components of AI methodologies—to interpret complex biological data.

Biomarker discovery leverages multi-modal integration of genomic, transcriptomic, and proteomic data. In immuno-oncology, the TIDE framework analyzes T-cell dysfunction and exclusion signatures to stratify immune checkpoint inhibitor responders [134]. CUPLR [135] and CUP-AI-Dx [136] further improve tissue-of-origin classification and microenvironment profiling, particularly in rare cancers. Multimodal AI models leverage variational autoencoders to jointly model gene expression, protein abundance, and methylation status, extracting latent representations predictive of clinical outcomes [137]. Similarly, MultiPLIER expands the PLIER framework to enable interpretable feature extraction across large transcriptomic cohorts like The Cancer Genome Atlas (TCGA) [138]. Another tool that should be mentioned is BABEL, which enables cross-modality translation by predicting gene expression from chromatin accessibility or protein abundance, allowing integrated analysis across scRNA-seq, ATAC-seq, and CITE-seq modalities [139].

Pharmacogenomic models that match tumor-specific mutations with drug sensitivity profiles have improved response rates [140]. Predictive platforms like Cox-nnet integrate neural networks with Cox regression to prioritize prognostic markers, improving five-year survival prediction [141]. SNP-informed pharmacogenomic tools are increasingly used in clinical oncology to tailor drug dosing and minimize toxicity risk [142]. Furthermore, liquid biopsy analysis using DL techniques now enables the detection of circulating tumor DNA (ctDNA) at extremely low allele frequencies, enabling early diagnosis and real-time monitoring of disease progression [143]. AI-enhanced tumor mutational burden (TMB) calculators have also demonstrated improved stratification of patients likely to benefit from immune checkpoint blockade [144,145].

### 4.5. Drug Discovery

AI has accelerated the process of identifying drug targets, repurposing existing drugs, and designing personalized therapies by leveraging high-throughput sequencing data. DL models now predict drug–target interactions with unprecedented accuracy [146], significantly accelerating preclinical development. Interestingly, transformer-based frameworks like MolTrans predict binding affinities directly from sequence and structural representations [147] (Table 2). These approaches have successfully identified previously overlooked targets when applied to cancer genomics datasets.

Drug repurposing has particularly benefited from AI integration in NGS data analysis. For instance, the DeepDR platform combines multiple drug-related networks—including transcriptomics, chemical structure, and disease association data—to identify candidates for existing medications, outperforming traditional repurposing strategies [148]. Another recent tool, MatchMaker, incorporates gene expression and chemical fingerprint data to identify cross-indication therapeutic candidates [149]. In precision oncology and rare disease contexts, pharmacogenomic models built from whole-exome sequencing can now predict optimal drug combinations with high accuracy. Platforms like DeepPurpose employ DL models to match genetic variants to compound libraries, expanding personalized treatment options [150] (Table 2). Furthermore, real-time NGS integration in adaptive clinical trials is now guiding therapy selection dynamically based on tumor evolution, improving patient stratification and trial efficiency [151].

## 5. AI-Driven Multi-Omics Integration and Clinical Translation

AI is becoming essential for leveraging multi-omics data and translating insights into clinical applications. By integrating genomics, transcriptomics, epigenomics, proteomics, and metabolomics, AI models uncover biological insights that transcend the limitations of single-omics approaches. Tools like OmiEmbed apply DL frameworks to capture shared biological variation across multiple omics layers, enabling a more comprehensive understanding of disease mechanisms [152].

Multi-omics integration strategies typically fall into three categories: early integration, where features from different omics are concatenated into a single input matrix; intermediate integration, which uses modality-specific encoders and combines latent representations; and late integration, where predictions from individual omics models are fused via ensemble methods. Among these, intermediate integration has become the most widely adopted in DL-based frameworks due to its flexibility and improved performance in heterogeneous datasets. Graph-based approaches have demonstrated strong performance in this space [153,154]. For example, MOGONET employs graph neural networks (GNNs) to learn patient similarity networks within each omics modality [155]. These graphs are then integrated through an attention-based mechanism that enhances classification and biomarker identification, particularly in cancer subtyping. Similarly, the integrative graph convolutional network (IGCN) builds sample-level graphs from high-dimensional input and applies convolutional operations to extract cross-omic interactions and shared regulatory patterns, facilitating accurate disease subtype discovery and survival prediction [156,157]. Despite these promising advances, several limitations remain. Multi-omics datasets often suffer from missing data, modality imbalance, and batch effects, which can distort model training. Additionally, the computational demands of training deep integration frameworks on high-dimensional inputs are substantial. Interpretability also poses a challenge, as black-box architectures may obscure biological mechanisms underlying predictions. Furthermore, the lack of standardized benchmarking datasets hinders rigorous comparisons between integration models, particularly in clinical contexts.

In clinical contexts, AI models are now central to personalized pipelines medicine. Platforms such as PathAI combine genomic, transcriptomic, histopathological, and clinical data to assist in disease diagnosis, predict patient outcomes, and suggest tailored therapeutic strategies, thereby accelerating data-to-decision workflows in precision oncology [158]. A major leap in variant interpretation is represented by DeepMind’s AlphaMissense, a ML model trained to predict the pathogenicity of missense mutations in human genomes [159] (Table 2). By analyzing protein structure and evolutionary data, the model assesses whether a given amino acid substitution is likely to cause disease, thereby providing functional annotations for variants of uncertain significance [160]. Despite these advances, translating AI tools into clinical practice presents significant challenges. Clinical validation requires extensive wet-lab confirmation, external dataset benchmarking, and diverse cohort representation to ensure model robustness. Regulatory approval remains complex, especially for “black-box” models where explainability and reproducibility are essential for trust and compliance with standards like FDA or CE marking. Furthermore, institutional inertia, integration with existing electronic health records (EHRs), and computational infrastructure gaps can delay real-world deployment. Nonetheless, tools like DeepVariant are being adopted in clinical diagnostic labs, and federated learning models are currently undergoing pilot testing in multi-center hospital networks, suggesting that regulatory and infrastructural paths are slowly being paved for AI-driven personalized medicine.

## 6. Challenges and Limitations

The integration of AI, ML, and DL into NGS analysis holds great promise, yet several challenges must be addressed to fully realize its transformative potential. First, the quality and quantity of sequencing data remain critical concerns. Technical artifacts, such as noise, amplification bias, and platform variability, can significantly affect model performance and reproducibility [121,161]. Moreover, limited sample sizes and class imbalance, particularly common in rare diseases and specific clinical cohorts, reduce model robustness and generalizability [157].

A major challenge lies in the interpretability of complex “black-box” models. Despite high predictive accuracy, the biological meaning of many AI outputs remains unclear. This lack of transparency hinders clinical adoption [162]. Explainable AI methods—such as SHAP analysis—are increasingly necessary to trace predictions back to interpretable molecular features [163]. Another significant issue is cross-platform variability. AI models trained on one sequencing technology often underperform when employed to datasets generated from other platforms [164]. While sequencing platform variability challenges model generalizability, the Protein Data Bank (PDB) provides a valuable example of successful cross-platform integration, standardizing protein structures resolved by X-ray crystallography, NMR, and cryo-EM through universal coordinate systems and file formats [165,166]. In contrast, a unified repository for DNA 3D structure remains lacking: although projects like the 4DN Data Portal compile uniformly processed datasets from Hi-C and related assays [167], and resources such as GSDB and 3DGD provide reconstructed 3D models from Hi-C, there is still no widely adopted standard file format or coordinate system for DNA architectures [168,169]. This absence of universal representation hinders AI model interoperability and cross-study validation and highlights the need for community-driven frameworks to support DNA structural genomics.

Data privacy and ethical concerns are of utmost importance in clinical and cross-institutional settings. Federated learning frameworks allow collaborative AI model development without centralized data sharing, thus preserving patient privacy while enhancing training data diversity [170]. These models are increasingly used in diagnostics and drug discovery to overcome institutional barriers to data access. Clinical translation of AI models remains an ongoing challenge. Even highly accurate predictions often require extensive wet-lab validation. Interpretability is crucial for gaining approval from regulatory authorities, especially in diagnostics [171]. Furthermore, population biases in training datasets can distort outcomes and limit applicability. Multimodal integration frameworks—combining methylation, transcriptomics, and chromatin accessibility—are helping address cell-type heterogeneity and improve signal resolution. Meanwhile, transformer-based models enable unified analysis across multiple omic layers, though clinical readiness requires more rigorous benchmarking [172,173]. In drug discovery, limited training data for novel or rare targets continues to constrain performance. Generative AI models show promise in designing new compounds from multi-omic profiles, and some AI-guided therapies are now entering clinical trials [174].

In summary, while AI has made remarkable progress critical challenges remain in data quality, interpretability, clinical validation, and privacy [175]. Continued collaboration between computational scientists, biologists, and clinicians will be key to navigating this evolving landscape.

## 7. Future Perspectives—AI Integration into Third-Generation Sequencing

Although NGS altered genomic research, it still presents numerous limitations due to the generation of short reads, which are surmounted with the advent of TGS. The revolutionary long-read technologies introduced by Pacific Biosciences^®^ (PacBio) and Oxford Nanopore Technologies^®^ (ONT) have transformed genome and transcriptome sequencing. TGS enables single-molecule, real-time sequencing and has introduced novel chemistries that produce long reads averaging 10 kb, compared to NGS’s 600 nt, thus allowing better analysis of complex genomic regions and improving the quality of the sequencing results [176]. By omitting the need for PCR amplification, TGS reduces biases that complicate computational analysis, allowing for faster, simpler, and more accurate decoding of nucleic acids [177]. Nevertheless, persistent challenges—such as high error rates and complex data interpretation—remain for both TGS and NGS. In this context, AI has become essential in addressing these limitations and enhancing data analysis.

AI was initially applied to the challenging yet crucial process of basecalling, enhancing the accuracy of converting raw signal data into nucleotide sequences. Notably, while early ONT basecallers (i.e., Metrichor) relied on hidden Markov models, later tools like Nanonet and DeepNano adopted recurrent neural networks [178,179]. The research basecaller Bonito combines CNNs with connectionist temporal classification (CTC) decoders [180], while the main ONT basecaller, Guppy, integrates CNNs with conditional random field (CRF) decoders [181]. In addition to ONT, AI has been also introduced to PacBio technology as errors in HiFi reads are reduced using DeepConsensus—a gap-aware sequence transformer encoder that produces high-accuracy consensus sequences [182].

While alignment of TGS data typically does not use AI approaches, variant calling increasingly relies on them. DL-based tools are gradually replacing traditional statistical methods to enhance the accuracy of long-read variant identification. Models like MAMnet and BreakNet, both CNN-based, predict indels from long-read data, while SVision is preferred for studying structural variants [183]. Other DL-based variant callers include Clairvoyante, Clair, and Nanocaller (pileup-based); PEPPER-Margin-DeepVariant (full alignment-based); Medaka (consensus-based); Clair3, which combines pileup and full-alignment algorithms [83]; and Clair3-RNA, a small variant caller for long-read RNA sequencing data for both ONT and PacBio platforms [184].

Of note, TGS is increasingly used to detect epigenetic modifications in DNA and RNA, offering many advantages over NGS. Recently, AI approaches have been developed to interpret the generated raw signal data that are crucial for this type of analysis. PacBio^®^ uses interpulse durations (IPDs) and pulse widths to infer DNA base modifications, while ONT detects modified bases through shifts in electrical current as DNA or RNA translocates through a nanopore. AI methods for identifying DNA modifications include Nanopolish, which uses HMMs to detect m5C; DeepSignal, which applies CNNs to detect m5C and m6A; and mCaller, a neural network classifier for identifying 6mA [180,185,186,187]. Additionally, SignalAlign combines HMMs with hierarchical Dirichlet process (HDP) models to correlate ionic current shifts with DNA modifications (m5C, hm5C, m6A) [188]. ONT’s direct RNA sequencing bypasses reverse transcription, enabling real-time detection of RNA modifications—an approach not possible with NGS. To analyze RNA methylation, MINES, a random forest classifier, assigns m6A to DRACH motifs [189], while EpiNano and Nanom6A focus on RRACH motifs [190,191]. Other tools, such as Nanocompore, use statistical models and ML for modified RNA base detection [192]; nanoDoc combines unsupervised DL with CNNs for RNA post-transcriptional modification analysis [193], and xPore, based on Bayesian deep learning, identifies differential RNA modification sites [194].

## 8. Conclusions

The integration of AI into NGS workflows has revolutionized genomics by enabling faster, more accurate, and scalable analysis of complex biological data. Through both ML and DL methods, AI facilitates tasks ranging from basecalling and variant calling to multi-omics integration, cancer research, and drug discovery. Notably, AI-based tools have demonstrated superior performance in detecting rare variants, reconstructing full-length transcripts, interpreting non-coding regions, and predicting treatment outcomes, especially in cancer genomics and single-cell studies. Ensemble and hybrid models further enhance prediction accuracy and generalizability by leveraging complementary algorithmic strengths. However, significant challenges remain in model interpretability, data heterogeneity, clinical validation, and privacy compliance. The success of AI applications in genomics depends not only on technical advancements but also on fostering interdisciplinary collaborations between data scientists, biologists, and clinicians. As long-read sequencing technologies and multi-modal datasets continue to evolve, AI will be pivotal in translating genomic insights into clinical practice. Future directions should prioritize the implementation of federated learning frameworks to preserve data privacy in cross-institutional AI training, the development of explainable AI models to enhance clinical trust and regulatory approval, and the seamless integration of TGS data to improve variant detection and epigenetic profiling. Robust benchmarking, regulatory validation pipelines, and interdisciplinary collaboration will be key to ensuring trustworthy AI deployment in diagnostic and therapeutic settings. Ultimately, AI promises to usher in a new era of precision medicine that is data-driven, individualized, and scalable.

## Figures and Tables

**Table 1:** Overview of Nextflow-based pipelines facilitating scalable and reproducible NGS workflows.

Pipeline	Application	Features	Reference
nf-core/circrna	circRNA and miRNA analysis	circRNA quantification, miRNA target prediction, differential analysis	[45]
nf-rnaSeqCount	RNA-seq quantification	QC, alignment, count quantification, MultiQC reporting	[46]
GeneTEFlow	Gene + TE expression	STAR/RSEM + SQuIRE quantification, DE analysis, Docker containerization	[47]
scATACpipe	scATAC-seq processing	Preprocessing, fragment/BED/BAM outputs, ArchR clustering, interactive HTML report	[48]
polishCLR	Genome assembly polishing	PacBio CLR polishing with Illumina data, haplotig purging, outputs quality-checked assemblies and logs	[49]
nf-gwas-pipeline	GWAS analysis	Automates genotype QC, population structure correction, association, and visualization	[50]
StellarPGx	Pharmacogenomics	CYP star allele calling, variant annotation, phasing and clinical interpretation	[51]
NFTest	Pipeline testing	Automated functional testing of Nextflow workflows using synthetic test cases	[52]
PoSeiDon	Positive selection and recombination analysis	Runs PAML CODEML for detecting positive selection and recombination in nucleotide alignments; configurable for HPC/Singularity deployments	[53]
Pipeliner	Sequencing data preprocessing	Modular processing for bulk and single-cell RNA-Seq; leverages Nextflow + Conda for reproducible workflows	[54]
FA-nf	Functional annotation	Nextflow-based functional annotation of novel genomes using Pfam, GO terms, database scaffolding	[55]
Geniac	Nextflow add-on	Auto-generates config files + containers; linter enforces standardized outputs	[56]
DolphinNext	Workflow management	GUI platform built on Nextflow; drag-and-drop pipeline design, monitoring, containerized reproducibility	[57]

**Table 2:** List of AI-based tools designed for NGS and TGS applications.

NGS/TGS Application	Tool/Platform	Functionality	Algorithm Type
**Variant Calling**	DeepVariant	SNP and indel calling	CNN
	DNAscope	High-accuracy genotyping	ML
	Clair3, Clairvoyante	Long-read variant calling	CNN
	Clair3-RNA	Small variant caller for long-read RNA sequencing data	DL
	PEPPER-Margin-DeepVariant	Alignment and consensus-based variant calling	DL
	ClinPred	Pathogenicity prediction for missense variants	Ensemble (XGBoost + RF)
	REVEL	Aggregated predictions for the pathogenicity of variant effects	Ensemble
	DeepFilter	Variant call filtering	DL
**Splicing Prediction**	SpliceAI	Splice-disruptive variants prediction	CNN
	Splam	Splice junctions in DNA prediction	DL/CNN
**Transcriptomics**	Bambu	Transcript discovery and quantification from long-read RNA-Seq data	ML
	Deep Count Autoencoder (DCA)	Gene expression denoising	DL
	scVI/scVAE	Batch correction, embedding, differential expression	DL
	MultiPLIER	Interpretable feature extraction across large transcriptomic cohorts	ML
	Cox-nnet	Patient prognosis prediction from high-throughput RNA-Seq data	ANN
	BABEL	Cross-modality translation between multi-omic profiles	DL
**Genomics**	DNABERT	Genome-wide prediction of promoters, splice sites, and transcription factor binding sites	Bidirectional encoder representation from transformers
**Methylation Analysis**	MethylNet	DNA methylation prediction	DL
	MethylSPWNet	Classification of CpGs into biologically relevant capsules	DL
	DeepMethyl	CpG methylation prediction	DL
	DiffuCpG	Methylation imputation	DL
	MethylGPT	Methylation value prediction	DL
**Chromatin Accessibility**	Basset	Prediction of accessible chromatin regions	CNN
	Enformer	Prediction of variant effects on gene expression	DL
	scATAC-pro	Quality assessment, analysis, and visualization of single-cell chromatin accessibility sequencing data	VAE
**Histone Modifications**	DeepHistone	Histone modification patterns prediction	NN/DL
	dHICA	Histone mark imputation and prediction of their modifications	DL
**Single-Cell Analysis**	CopyKAT	CNV inference from scRNA-seq	Integrative Bayesian segmentation approach
	scGraphformer	Unveiling cellular heterogeneity and interactions in scRNA-seq data	Transformer-based GNN
	TotalVI	Multi-modal data analysis/joint analysis of CITE-seq data	VAE
	scANVI	Cell state/transcriptomics data annotation	DL
	scShaper	Accurate linear trajectory inference	Ensemble
**3D Genome Structure**	ChromoGen	Single-cell chromatin conformation modeling	Generative model
**Cancer Research**	PyClone-VI	Inference of clonal population structures using whole genome data	Bayesian statistical method
	HoneyBADGER	Identification of CNVs and heterozygosity loss in individual cells from single-cell RNA-Seq	HMM-integrated Bayesian hierarchical model
	CUPLR	Tissue of origin classification for cancer of unknown primary diagnostics	ML
	CUP-AI-DX	Inference of cancer tissue of origin and molecular subtyping using gene expression data	CNN
**Drug Discovery**	DeepDR	Drug repurposing	DL
	DeepPurpose	Drug–target interaction prediction	DL
	MolTrans	Drug–target interaction prediction	DL
	MatchMaker	Drug synergy prediction	DL
**Clinical Diagnostics**	AlphaMissense	Variant pathogenicity prediction	ML
**Multi-Omics Integration**	OmiEmbed	Multi-omics data analysis	DL
	MOGONET	Patient classification and biomarker identification	GNN

## Data Availability

Not applicable.

## Abbreviations

The following abbreviations are used in this manuscript:

AI	Artificial intelligence
ANN	Artificial neural network
ATAC-seq	Assay for transposase-accessible chromatin using sequencing
AUC	Area under the curve
CITE-seq	Cellular indexing of transcriptomes and epitopes by sequencing
CNN	Convolutional neural network
CNV	Copy number variation
CRF	Conditional random field
CTC	Connectionist temporal classification
ctDNA	Circulating tumor DNA
DL	Deep learning
DEA	Differential expression analysis
DMR	Differentially methylated region
EMBL-EBI	European Bioinformatics Institute
GATK	Genome Analysis Toolkit
GBM	Gradient boosting machine
GEO	Gene Expression Omnibus
GNN	Graph neural network
GRN	Gene regulatory network
HDP	Hierarchical Dirichlet process
HGP	Human Genome Project
HMM	Hidden Markov model
IGCN	Integrative graph convolutional network
IPD	Interpulse duration
LSTM	Long short-term memory
ML	Machine learning
m5C	5-methylcytosine
m6A	N6-methyladenosine
hm5C	5-hydroxymethylcytosine
ML	Machine learning
mRNA	Messenger RNA
NGS	Next-generation sequencing
ONT	Oxford Nanopore Technologies
PCA	Principal component analysis
PCR	Polymerase chain reaction
RF	Random forest
RNN	Recurrent neural network
scRNA-seq	Single-cell RNA sequencing
SdAs	Stacked denoising autoencoders
SNP	Single nucleotide polymorphism
SVM	Support vector machine
TCGA	The Cancer Genome Atlas
TGS	Third-generation sequencing
t-SNE	t-distributed stochastic neighbor embedding
UMAP	Uniform manifold approximation and projection
VAE	Variational autoencoder
WGBS	Whole-genome bisulfite sequencing
